# Biomarkers and clonality before and after treatment

**DOI:** 10.1186/1742-4690-11-S1-O31

**Published:** 2014-01-07

**Authors:** Juan Carlos Ramos

**Affiliations:** 1 Division of Hematology-Oncology, Department of Medicine, Sylvester Comprehensive Cancer Center, University of Miami, Miami, FL, USA

## 

Adult T-cell leukemia-lymphoma (ATLL) is a disease with dismal prognosis urging new therapies. Few biomarkers have been identified to predict disease outcome in ATLL, however, routine testing for these is not widely available. The first line treatment option for ATLL can vary according to disease subtype, investigator’s choice, or institutional practices. For instance, experience at some centers suggest that there are patients with ATLL who can benefit from zidovudine (AZT)/interferon alpha (IFNα) therapy without requiring chemotherapy upfront. It has been reported that p53 gene alterations or MUM-/IRF-4 expression status may influence response to AZT/IFNα. More recently, the use of the anti-CD30 monoclonal antibody brentuximab vedotin is being advocated for the treatment of CD30+ T-cell malignancies, including ATLL. Therefore, establishing biomarkers that can help guide therapeutic decisions for ATLL would be ideal. Updated results and the usefulness of testing for some of these biomarkers will be discussed.

